# Are systolic function and ejection fraction interchangeable? New insights from cardiovascular magnetic resonance and in-vivo validation of mathematical models of LV function

**DOI:** 10.1186/1532-429X-17-S1-P325

**Published:** 2015-02-03

**Authors:** Jonathan C Rodrigues, Stephen Rohan, Amardeep Ghosh Dastidar, Amy E Burchell, Laura E Ratcliffe, Emma C Hart, Angus K Nightingale, Julian F Paton, Chiara Bucciarelli-Ducci, Mark Hamilton, Nathan E Manghat, David H MacIver

**Affiliations:** 1 CMR Unit, NIHR Cardiovascular Biomedical Research Unit, Bristol Heart Institute, Bristol, UK; 2 School of Physiology and Pharmacology, The University of Bristol, Bristol, UK; 3 Medical School, The University of Bristol, Bristol, UK; 4 Cardionomics Research Group, Bristol Heart Institute, Bristol, UK; 5 Cardiology, Taunton and Somerset Hospital, Taunton, UK; 6 Biological Physics, The University of Manchester, Manchester, UK

## Background

Clinical studies have shown that many patients may have abnormally reduced myocardial shortening despite preserved ejection fraction (EF). Mathematical modeling explains this apparent paradox by demonstrating, that whilst myocardial shortening determines radial strain (RS), it is absolute wall thickness (AWT) rather than RS that determines EF. We sought in vivo cardiac MR (CMR) evidence to support the mathematical modeling theories.

## Methods

We retrospectively analysed 39 CMR studies (28 hypertensive patients and 11 healthy volunteers [mean age: 47.4±13.0, male: 56.4%]) performed at 1.5T (Avanto, Siemens). Ventricular volumes and EF were measured using established CMR techniques. Left ventricular wall thickness was measured at end-diastole (ED) and end-systole (ES) in the long- and short-axis cine views at the mid-cavity at the level of the papillary muscles. Measurements were repeated twice, 1 day apart. Longitudinal strain (LS) was estimated using a modified 6-point mean annular plane systolic excursion of the mitral valve from 3-chamber, 2-chamber and 4-chamber cines. Subgroup analysis was performed by EDWT (Group 1 <9mm [n= 19] and Group 2 >9mm [n=20]). Continuous variables were compared by Student *t* tests and categorical variables by Fisher exact test (setting *p*<0.05 as significant).

### Definitions

AWT = ES thickness - ED thickness.

RS = (AWT / ED thickness) x 100.

Midwall circumferential fractional shortening (mFS) = ((LVIDd+EDWT)-(LVIDs+H))/(LVIDd+EDWT)x100 (%), where H = ((LVIDd+EDWT)^3^-(LVIDd)^3^+(LVIDd)^3^/^1/3^-LVIDs, LVIDd = left ventricular internal dimension in diastole, LVIDs = left ventricular internal dimension in systole and EDWT = end diastolic wall thickness.

## Results

Intra-observer variability for wall thickness measurements was good both both long-axis and short-axis measurements (intra-class correlation coefficient=0.931).

**Figure 1:**
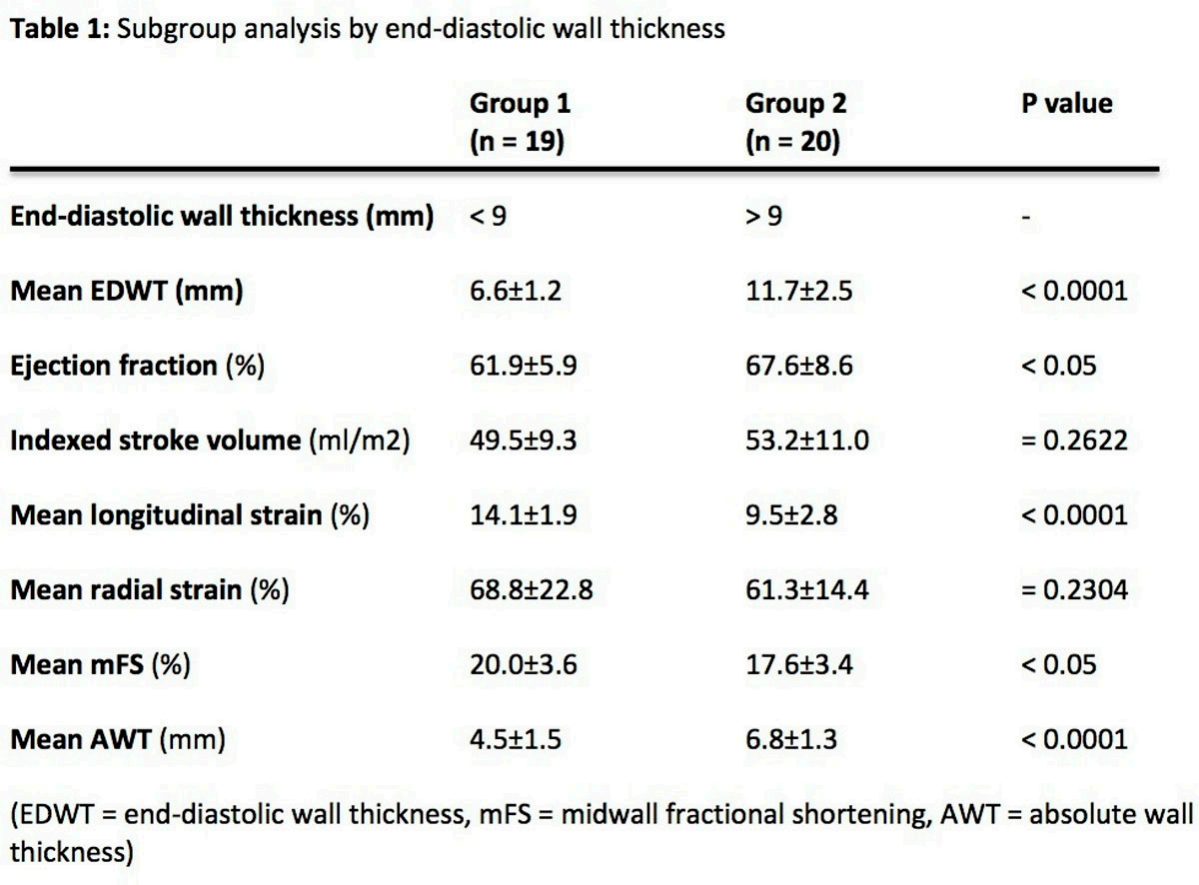
Subgroup analysis by end-diastolic wall thickness

Significant positive correlation was demonstrated between EDWT and AWT (r^2^=0.42, p<0.0001). However, significant negative correlations were demonstrated between EDWT and RS (r^2^=0.13, p<0.05), between EDWT and longitudinal strain (r^2^=0.62, p<0.0001) and between EDWT and mFS (r^2^=0.16, p<0.05). Subgroup analysis by EDWT is demonstrated in Figure 1. Despite significant reductions in LS, mFS and trend towards lower RS, ejection fraction is significantly higher and indexed stroke volume is similar between in ventricles with EDWT >9mm compared to those <9mm.

## Conclusions

Myocardial systolic function and ejection fraction are not the same. Our study provides in-vivo validation of mathematical modeling predictions that significant LV systolic impairment (in the form of impaired LS, RS and mFS) can be compensated for by significant increases in AWT thus maintaining indexed SV and EF. This finding has implications for the understanding the pathophysiology of heart failure with preserved ejection fraction, and its potential treatment.

## Funding

NIHR Bristol Cardiovascular Biomedical Research Unit, Bristol Heart Institute.

JCLR: Clinical Society of Bath Postgraduate Research Bursary.

ECH: BHF grant IBSRF FS/11/1/28400.

